# Three novel beta-galactosidase gene mutations in Han Chinese patients with GM1 gangliosidosis are correlated with disease severity

**DOI:** 10.1186/1423-0127-17-79

**Published:** 2010-09-30

**Authors:** Chi-Fan Yang, Jer-Yuarn Wu, Fuu-Jen Tsai

**Affiliations:** 1 Institute of Biomedical Sciences, Academia Sinica, Academia Road, Nankang, Taipei, Taiwan; 2 Graduate Institute of Chinese Medical Science, China Medical University, Hsueh-Shih Rd, Taichung, Taiwan; 3 School of Post-baccalaureate Chinese Medicine, China Medical University, Hsueh-Shih Rd., Taichung, Taiwan; 4 Department of Medical Genetics, Pediatrics and Medical Research, China Medical University Hospital, Yuh-Der Rd., Taichung, Taiwan; 5 Department of Biotechnology and Bioinformatics, Asia University, Liufeng Rd., Wufeng, Taichung, Taiwan

## Abstract

**Background:**

GM1 gangliosidosis (GM1) is an autosomal recessive lysosomal storage disease caused by deficiency of acid beta-galactosidase (GLB1; EC3.2.1.23). Here, we identify three novel mutations in the GLB1 gene from two Han Chinese patients with GM1 that appear correlated with clinical phenotype.

**Methods:**

One of the two Han Chinese patients with GM1 presented with the juvenile form, and the other with the infantile form with cardiac involvement. Sequencing of the entire GLB1 gene revealed three novel mutations (p.H102 D, p.G494V, c.495_497delTCT), which were absent in 94 normal controls. Transient expression of cDNA encoding these variants was performed in COS-1 cells to evaluate β-galactosidase activities.

**Results:**

The first case (patient 1) with the juvenile form contained two missense mutations, p.H102 D and p.A301V. Patient 2 diagnosed with the infantile form of the disease with cardiac involvement was compound heterozygous for p.G494V and c.495_497delTCT mutations. All mutant beta-galactosidases exhibited significantly reduced activity (12%, 0%, 0%, and 0% for p.H102 D, p.A301V, p.G494V, and c.495_497delTCT), compared with the wild-type beta-galactosidase cDNA clone. The mutations identified in patient 2 with cardiomyopathy were localized in the GLB1 gene region common to both lysosomal beta-galactosidase and elastin binding protein (EBP), and caused a deletion in the elastin-binding domain of EBP.

**Conclusions:**

All four mutations identified in Han Chinese patients induce significant suppression of β-galactosidase activity, correlating with severity of disease and presence of cardiomyopathy.

## Introduction

Human β-galactosidase (E.C.3.2.1.23; MIM# 230500) is an lysosomal enzyme that removes β-ketosidically linked galactose residues from glycoproteins, sphingolipids, and keratin sulfate [1]. Deficiency of acid β-galactosidase leads to two metabolic storage diseases, specifically, GM1 gangliosidosis (GM1) and Morquio B disease (MBD, mucopolysaccharidosis type IVB, MPS IVB), inherited as autosomal recessive traits. In GM1 gangliosidosis, absence or suppression of β-galactosidase activity causes excessive accumulation of GM1 ganglioside in neuronal tissue while β-linked galactose-terminal oligosaccharides arising from the lysosomal digestion of glycoproteins are stored in visceral organs and excreted in urine. Three major clinical phenotypes have been classified to date: infantile (type I), late infantile/juvenile (type II), and adult (type III) forms [1]. The most severe infantile form with onset between birth and 6 months results in rapidly progressive central nervous system (CNS) degeneration, visceromegaly, cherry-red spots, skeletal abnormalities, and death usually occurring within the first two years of life. Cardiomyopathy is an atypical clinical feature in some Caucasian patients with infantile GM1 gangliosidosis. The residual β-galactosidase activity in fibroblasts from patients is less than 1.3%, compared to the physiological level. However, the juvenile and adult forms of GM1 gangliosidosis display a less severe course, later age of onset, and higher β-galactosidase activity, varying from 0.3 to 4.8% of the normal level in the juvenile form and ~9% in the adult form [1,2].

In contrast to GM1 gangliosidosis, Morquio B disease (MBD) is characterized by severe skeletal dysplasia without CNS involvement. MBD is also associated with deficiency of acid β-galactosidase, but the metabolic storage substance in this case is keratin sulfate, a glycosaminoglycan accumulating in the cornea and skeletal tissue and secreted in urine, and not GM1 ganglioside in the brain [1].

The human β-galactosidase gene (GLB1) located on chromosome 3p21.33 [3] contains 16 exons spanning approximately 62.5 kb [3,4]. The GLB1 gene encodes two alternatively spliced products, lysosomal β-galactosidase (GLB1) and elastin-binding protein (EBP) [5-7]. The GLB1 protein is synthesized as an N-glycosylated 85 kDa precursor, and processed within lysosomes to a 64 kDa mature enzyme [8-10]. The active enzyme forms complexes with the 54 kDa protective protein/cathepsin A (PPCA) and the 46 kDa neuraminidase (NEU1) in lysosomes. PPCA is essential for intracellular transport and intralysosomal activity/stabilization of both β-galactosidase and NEU1 [11,12]. NEU1 catalyzes hydrolysis of the terminal sialic acids of oligosaccharides, glycoproteins and glycolipids [13,14]. The alternative splice product, EBP, results from deletion of exons 3, 4 and 6, and a frameshift in the translation of exon 5 encoding the unique elastin binding domain of EBP [5,15].

Cardiomyopathy in GM1 gangliosidosis is associated with impaired elastogenesis and EBP defects [16-19]. EBP, a 67 kDa enzymatically inactive variant of β-galactosidase, is not targeted to lysosomes. Earlier studies show that EBP acts as a recycling chaperone that protects tropoelastin from premature degradation and delivers it through an intracellular secretory pathway to the cell surface where tropoelastin is secreted into the extracellular matrix and assembled [6,20-22]. Tropoelastin is released from EBP following a conformational change that occurs after the galactolectin domain of EBP binds to galatosugars protruding from glycoproteins on the microfibrillar scaffold of growing elastic fibers [6,21]. Following tropoelastin release, EBP returns to the endosomes, binds to newly synthesized tropoelastin in the trans-Golgi network, and delivers it to the cell surface [23]. EBP additionally forms complexes with PPCA and NEU1 to facilitate elastic fiber assembly on the cell surface [24]. PPCA appears to act as a protective protein, while NEU1 is required for the release of tropoelastin and the signaling pathway induced by the complex [25,26].

To date, More than 130 mutations have been identified in the GLB1 gene [2,27,28]. In this study, we investigate the GLB1 mutations associated with GM1 gangliosidosis in two Han Chinese patients. Three of the mutations identified in these two patients were novel and have not been reported in patients of other ethnicities. We further evaluate the contribution of individual mutations to the clinical phenotype via site-directed mutagenesis experiments, examination of expression levels in COS-1 cells, and β-galactosidase activity measurements. One of the patients (patient 2) with cardiac involvement presented two mutations likely to affect both lysosomal GLB1 and EBP proteins, including a deletion in the elastin-binding domain of EBP. Our findings suggest a correlation between these mutations and the presence of cardiomyopathy.

## Patients and Methods

### Patients

The most important clinical features of two GM1 gangliosidosis patients are compared in Table 1. Patient 1, a female, was born after a normal pregnancy. She was the second child of non-consanguineous healthy parents, and the family history was non-contributory. The patient appeared normal at birth. At 6 months of age, abdominal distention, and subsequently, progressive developmental regression, delayed psychomotor development, and recurrent pulmonary infection were observed. At the age of 18 months, the patient displayed coarse facial features, thick skin with Mongolian spots, macroglossia, hepatosplenomegaly, scoliosis, and progressive leukomalacia. MR imaging revealed myelination arrest in the subcortical white matter, and cortical atropy. Enzyme assay of cultured fibroblasts disclosed 2% residual activity compared to the normal level. The patient's clinical condition deteriorated rapidly, and she died of severe emaciation and hypovolemic shock at 2 years, 2 months of age. Based on residual β-galactosidase activity, onset age, disease progression and age at death, the patient was diagnosed with the juvenile form of the disease [1,2].

**Table 1:** Clinical and molecular features of two Taiwanese patients with GM1 gangliosidosis

	Patient 1	Patient 2
Phenotype	Juvenile	Infantile
Age of onset	6 months	Birth
Age at diagnosis	18 months	4 months
Presentation	Developmental regression, delayed psychomotor development, coarse facial features, macroglossia, abdominal distention, and thick skin with Mongolian spots	Developmental regression, hypotonia, bilateral hydrocele, and visceromegaly
Eye	Normal	Cherry red spot
Heart	Normal	Dilated cardiomyopathy, Hypertrophied left atrium/ventricle, and poor contractility of left ventricle
Skeleton	Scoliosis and beaking of the lumbar vertebral bodies	Normal
Liver/spleen	Hepatosplenomegaly	Hepatomegaly
Nervous system	White matter demyelination and cortical atropy	White matter demyelination, cortical atropy, and seizure
Bone marrow	Foamy histiocytes and progressive leukomalacia	Foamy histiocytes
Age of death	2 years, 2 months	2 years
β-galactosidase activity	2% of control value in fibroblast	< 1% of control value in fibroblast
Genetic defect		
Allele 1	c.304C > G (p.H102 D, inherited from father)	c.495_497delTCT (p.L166del, inherited from mother)
Allele 2	c.902C > T (p.A301V, inherited from mother)	c.1481G > T (p.G494V, inherited from father)
Polymorphisms	p.L10P	p.L10P

Patient 2 presented with the infantile form of the disease, and was the first child of a healthy non-consanguineous couple with no contributory family history. He had developmental retardation, and visceromegaly developed soon after birth. At 3 months of age, the patient presented with macular cherry-red spots and hepatomegaly. Hypotonia and bilateral hydrocele were noted at 4 months of age. The roentgenogram and echocardiogram findings included cardiomegaly, dilated cardiomyopathy, hypertrophied left atrium/ventricle, and poor contractility of the left ventricle. Residual β-galactosidase activity was lower than 1% of the normal level. MR imaging at 3, 12 and 23 months revealed significant myelination arrest with dysmyelination of white matter in the bilateral cerebral hemispheres and atropic change of the brain cortex [29]. The patient died of congestive heart failure and seizure at 2 years of age.

Healthy controls were randomly selected from the Taiwan Han Chinese Cell and Genome Bank [30]. All participating patients and controls were Han Chinese, which is the origin of 98% of the Taiwanese population. The study was approved by the institutional review board and the ethics committee of each institution. Written informed consent was obtained from participants, in accordance with institutional requirements and the Declaration of Helsinki Principles.

### Mutation analysis

Genomic DNA was isolated from peripheral blood or fibroblasts using standard techniques. We sequenced all exons of the β-galactosidase gene (GLB1), including the 5' and 3' untranslated regions and intron/exon boundaries. PCR primers were designed using the Primer 3 program http://frodo.wi.mit.edu/cgi-bin/primer3/primer3_www.cgi. Amplified products from genomic DNA were purified from the agarose gel using QIAEX II (Qiagen, Hilden, Germany), and directly sequenced in both directions using a BigDye Terminator cycle sequencing kit with an ABI Prism 377 DNA Sequencer (Applied Biosystems, Foster City, CA). The GLB1 genomic sequence was derived from GenBank, NT_022517.16. Nucleotide numbering was based on the cDNA reference sequence from GenBank, NM_000404.2, according to the journal guidelines http://www.hgvs.org/mutnomen, with A of the ATG start codon as +1.

### Construction of the GLB1 expression plasmid, pGS3

Total RNA was extracted from a normal lymphocyte cell line, and cDNA synthesized using the Advantage RT-for-PCR kit (Clontech, Palo Alto, CA) with an oligo-dT primer. Amplification of wild-type GLB1 gene was performed with the following oligonucleotides: forward, 5'-GTCATGCCGGGGTTCCTGGTTC-3', and reverse, 5'-GTCCCTGAAGGTGGGGCTTTGG-3', using the Expand™ High Fidelity PCR system (Boehringer Mannheim, Indianapolis, IN). The 2.21 kb PCR product was ligated into the TOPO TA cloning kit vector (Invitrogen, Carlsbad, CA), and the nucleotide sequence and orientation of the insert in the resulting plasmid, pTA-GS, verified by direct sequencing. The *Hin*dIII-*Xba*I fragment of pTA-GS was further subcloned into the expression vector, pcDNA3.1 (Invitrogen, Carlsbad, CA), generating the GLB1 expression plasmid, pGS3.

### Construction of mutant β-galactosidase expression plasmids with deletion and missense mutations

Site-directed mutagenesis using the recombinant polymerase chain reaction method [31] was performed to introduce the p.His102Asp, c.495_497delTCT, p.Ala301Val, and p.Gly494Val mutations into wild-type GLB1 cDNA. Wild-type pGS3 was employed as the template, and the primers used for site-directed mutagenesis are presented in Table 2. Nucleotide sequences of the resulting mutant GLB1 expression plasmids, pGS3-H102 D, pGS3-c.495_497delTCT, pGS3-A301V and pGS3-G494V, were confirmed for the entire coding region via direct sequencing.

**Table 2:** Primers used for site-directed mutagenesis

Genetic variation	Amino acid change	Exon	Oligonucleotides (5' -> 3')
c.304C > G	p.His102Asp	3	F: AACTGGTACTGTCCTGGCCAGGGCTCATGAAAGTTCCAG
			R: CCTGGCCAGGACAGTACCAGTTTTCTGAGGACGATGATGTGGAATATTTTC
c.495_497delTCT	p. L166del	5	F: ACCACTTGTCCACAGCTGCCAGGTAATCTGGGTCGGAGG
			R: CTGGCAGCTGTGGACAAGTGGTTGGGAGTCCT---GCCCAAGATGAAGCCTC
c.902C > T	p.Ala301Val	8	F: AAGTATATCATAGAGGGAGGAAGCCACTGCTTCGGTCTTG
			R: TTCCTCCCTCTATGATATACTTGCCCGTGGGGTGAGTGTGAACTTGTACATG
c.1481G > T	p.Gly494Val	15	F: GTTGATATATGCACCATAGTTCACACGTCCCATGTTCTC
			R: GAACTATGGTGCATATATCAACGATTTTAAGGTTTTGGTTTCTAACCTG

^1^Nucleotide changes are marked with an underline (nucleotide substitution) or a string of hyphens (deletion).

### Transient transfection of COS-1 cells

COS-1 cells were cultured in DMEM and 10% fetal bovine serum supplemented with 100 U/ml penicillin, 100 μg/ml streptomycin and 2 mM L-glutamine. Cells were grown to 85-90% confluency in each well of a 6-well plate, and separately transfected with 4 μg of pGS3-H102 D, pGS3-c.495_497delTCT, pGS3-A301V, pGS3-G494V and wild-type pGS3. Transfection was performed using the Lipofectamine™ 2000 reagent (Gibco BRL, Grand Island, NY), according to the manufacturer's instructions. After 48 h incubation at 37°C, cells were scraped and washed twice with PBS. Cell pellets were frozen at -70°C until use for protein content determination and assay of enzyme activity.

### Enzyme assays

Transfected cells were resuspended in distilled water, and subjected to sonication using a Misonix Ultrasonic processor (XL2020) at a power level of 10 for 15 s in ice. Soluble lysates were collected and protein concentrations determined using the Bio-Rad assay kit (Hercules, CA) with bovine serum albumin as a standard. The GLB1 enzyme assay was performed using the artificial substrate, 4-methylumbelliferyl-β-D-galactopyranoside (Sigma, St. Louis, MO) [32,33]. Briefly, β-galactosidase activity was measured in duplicate by adding 100 μl of enzyme solution to 300 μl of 0.65 mM substrate solution (4-methylumbelliferyl-β-D-galactopyranoside in 0.1 M citrate-phosphate buffer, pH 4.2). After incubation at 37°C for 1 h, all reactions were terminated by adding 1 ml of 0.1 M 2-methylpropan-1-ol, pH 10. Fluorescence was determined with a PerSeptive Biosystems (Cytofluor^® ^series 4000) fluorescence multi-well plate reader (Applied Biosystems, Foster City, CA). A known amount of 4-methylumbelliferone (Sigma, St. Louis, MO) in 0.1 M 2-methylpropan-1-ol, pH 10, was employed as the standard.

## Results

### Molecular analysis of GM1-gangliosidosis patients

**Figure 1:**
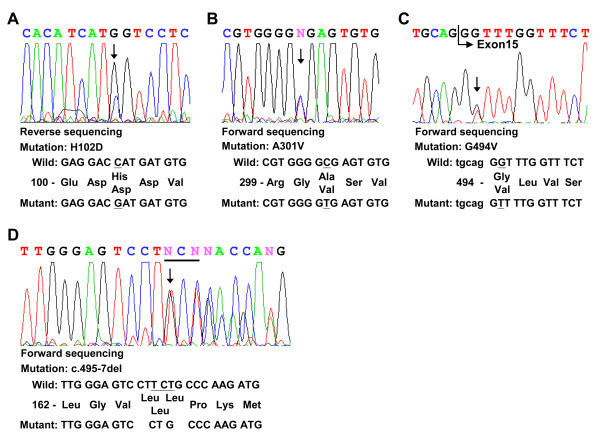
**Nucleotide sequences of the neighboring regions of the mutations in the GLB1 gene of two GM1 gangliosidosis patients**. The mutant sequencing diagrams are shown. The position of each mutation is marked with an arrow (panels A-D). Wild-type and mutant nucleotide and amino acid sequences are presented using A of the ATG start codon of GLB1cDNA as position +1.

**Figure 2:**
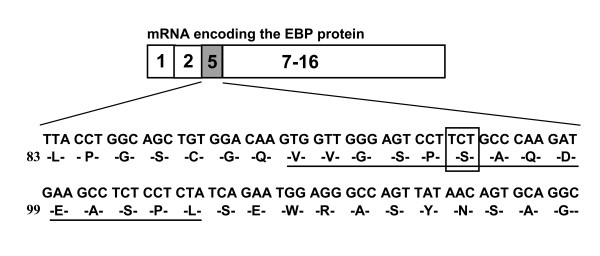
**Schematic representation of alternatively spliced mRNA of the GLB1 gene encoding EBP**. The unique 32 amino acid region encoded by a frameshift in exon 5 contains an elastin/laminin-binding domain (underlined). The 3 bp in-frame deletion resulting in p.S95del is enclosed within a square.

**Figure 3:**
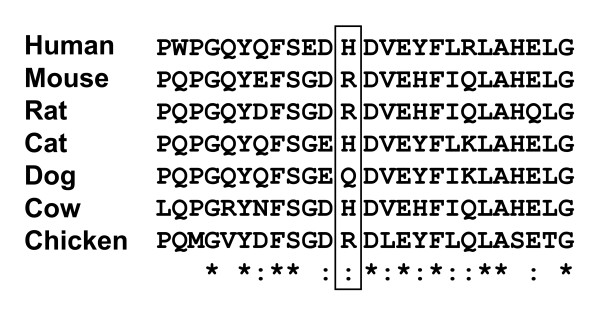
**Alignment of the GLB1 homologs of six species around the H102 D variants indicated by squares http://www.ebi.ac.uk/Tools/clustalw/index.html**. *Total sequence homology;: Very high homology;. High homology.

Genomic DNA from two Han Chinese patients with GM1 was amplified with a view to screening all 16 exons and splice junctions of the GLB1 gene via direct sequencing. In total, one deletion, three missense mutations and one polymorphism were identified (Tables 1 and 2). Among these, three mutations (p.H102 D, p.G494V, and c.495_497delTCT) were novel. Patient 1 diagnosed with the juvenile form of GM1 contained two mutations, specifically, c.304C > G in exon 3 (p. H102D) and c. 902C > T in exon 8 (p. A301V)[28] (Fig.1A and 1B), as well as one polymorphism in exon 1, homozygous p.L10P polymorphism (rs.7637099, data not shown). Patient 2 presenting with the infantile form of the disease contained a 3 bp in-frame deletion in exon 5, resulting in deletion of a residue, both in the GLB1 protein (c.495_497delTCT, p. L166del, Fig. 1D) and elastin-binding domain of the EBP protein (c.283_285delTCT, p.S95del, Fig.2). Another nucleotide mutation, c.1481G > T in exon 15, led to the generation of p.G494V (Fig. 1C). Patient 2 was heterozygous for the p.L10P polymorphism (data not shown). These variations in the GLB1 gene were confirmed in parent genomic DNA (Table 1). The p.L10P polymorphism was identified in the 94 normal Han Chinese subjects at an allele frequency of 47.3% (87 of 184 alleles) while the p.His102Asp, c.495_497delTCT (p.Leu166del), p.Ala301Val, and p.Gly494Val variants were absent in the 94 normal Han Chinese subjects. Alignment of the GLB1 sequences from human, mouse, rat, cow, cat, dog, and chicken revealed that p.Leu166, Ala301, and Gly494 are conserved throughout these species. All residues corresponding to human p.His102 among the species examined are basic (histidine, arginine or glutamine) whereas mutant p.Asp102 contains an acidic residue (Fig. 3). Gene variants were further analyzed for functional consequences on GLB1 enzyme activity in COS-1 cells.

### Transient expression of GLB1 plasmids in COS-1 cells

Expression patterns of these variant constructs in COS-1 cells were compared to those transfected with the normal GLB1 clone, pGS3 (Table 3). The GLB1 enzyme activities of cells transfected with pGS3-H102 D, pGS3-c.495_497delTCT, pGS3-A301V, and pGS3-G494V were 12%, 0%, 0%, and 0%, compared to those transfected with the control plasmid (pGS3), respectively.

**Table 3:** β-Galactosidase activity measured in transiently transfected COS-1 cell lysates

Vector name	Specific Activity (nmol/hr/mg of protein)^1^	Percentage of wild-type activity^2^
Controls		
None	320 ± 21.1	0
pGS3 wt	534 ± 27.7	100
		
Variants		
pGS3-H102D	346 ± 19.4	12
pGS3-c.495_497delTCT	237 ± 14.5	0
pGS3-A301V	260 ± 20.3	0
pGS3-G494V	294 ± 27.5	0

^1^Enzyme activities are presented as average values of two independent experiments, as described in the Patients and Methods section.

^2^Specific activities were obtained after subtraction of activity in mock-transfected COS-1 cells measured as a baseline control in each experiment.

## Discussion

More than 130 mutations in the GLB1 gene have been reported in GM1 or MBD cases from Japan, Northern/Southern America, and Europe. The majority of mutations appear to cluster in exons 2, 6, and 15 of the GLB1 gene [2]. These "hotspot" regions play important catalytic and/or regulatory roles. Here, we perform mutational analysis of two Han Chinese patients with GM1. Clinical manifestations of the patients can be distinguished according to the presence or absence of cardiac involvement. This atypical clinical feature has been reported in limited Caucasian patients. Three mutations in the GLB1 gene identified from the entire pathogenic allele of these two patients have not been reported previously. The variable severity of these clinical manifestations correlates with the mutation combinations. Patient 1, diagnosed with juvenile GM1 gangliosidosis without cardiomyopathy, is compound heterozygous for p.H102 D in exon 3 and p.A301V in exon 8, respectively. Although substitution of valine at position 301 with alanine seems to be homologous and non-deleterious, COS-1 cells expressing cDNA encoding p.A301V *in vitro *displayed no GLB1 enzyme activity. Low enzyme activity (~12% of the physiological level) was detected in COS-1 cells transfected with cDNA encoding the p.H102 D substitution. The p.H102 D appears to be a mild mutation responsible for the less severe phenotype of our juvenile patient displaying 2% residual enzyme activity. The p.A301V has been found in our patient 1 and in another juvenile patient [28], both presenting juvenile GM1 with central nervous system involvement and skeletal affection as well as absence of cardiac dysfunction and cherry red spots. The p.H102 D alone or combined with p.A301V can be related to hepatosplenomegaly.

Both c.495_497delTCT and p.G494V mutations identified in patient 2 led to absence of GLB1 enzyme activity in transient expression studies, correlating with lack of *in vivo *residual enzyme activity (< 1%). Two transcripts are processed from the primary β-galactosidase transcript, specifically, the classical lysosomal form of 677 amino acid β-galactosidase (GLB1) and the elastin binding protein (EBP) containing 546 residues in which exons 3, 4, and 6 are spliced out from pre-mRNA and exon 5 is frameshifted [5]. The frameshift in exon 5 results in a unique form of EBP, a 32-residue sequence containing an elastin/laminin-binding domain [15]. The two heterozygous mutations identified in patient 2 displaying cardiac involvement are localized in the GLB1 gene region common to the two transcripts, and thus affects both the lysosomal GLB1 enzyme and EBP protein. Both mutations can be related to infantile GM1 with cardiac dysfunction, as well as central nervous system involvement, cherry red spots and lack of skeletal affection.

The c.495_497delTCT mutation of the GLB1 transcript corresponds to a c.283_285delTCT variation of the EBP transcript, leading to in-frame deletion of an amino acid, p.Ser95del, in the elastin/laminin-binding domain of EBP. This variation in the elastin-binding domain of the EBP protein may directly impair tropoelastin binding to EBP and assembly of elastic fibers, which are consequently linked to the patient's cardiac involvement. Our data additionally support an association of EBP with cardiac involvement in GM1 gangliosidosis [16-19].

## Conclusions

We identified three novel genetic mutations in the GLB1 coding region from Chinese patients with GM1. Patient 1 presented with the juvenile form of GM1 and 2% residual β-galactosidase activity in cultured fibroblasts. Expression of the two GLB1 mutants identified in patient 1, p.H102 D and p.A301V, in COS-1 cells led to 12% and 0% of normal activity. Patient 2 presented with the infantile form of GM1 and less than 1% of residual β-galactosidase activity in cultured fibroblasts. The two mutants, p.G949V and c.934-7del (p.L166del), identified from patient 2 were devoid of activity upon expression in COS-1 cells. The two mutations in patient 2 were localized in the GLB1 gene region common to lysosomal GLB1 and EBP, and could therefore affect both proteins. All the mutations correlated with the clinical manifestations of patients and residual β-galactosidase activity in cultured fibroblasts. While elastic fiber deposition requires further evaluation in patients, it is plausible that impaired elastogenesis and cardiomyopathy in GM1 gangliosidosis are caused by defects in the elastin-binding domain of EBP.

## Abbreviations

GM1: GM1 gangliosidosis; GLB1: beta-galactosidases; EBP: elastin binding protein; PPCA: protective protein/cathepsin A; NEU1: neuraminidase.

## Competing interests

The authors declare that they have no competing interests.

## Authors' contributions

JYW conceived and designed the study. CFY performed molecular analysis and transient expression, and drafted the manuscript. FJT participated in the design of the study and critically reviewed the draft. All authors have read and approved the final manuscript.
